# High-risk type human papillomavirus infection and p16 expression in laryngeal cancer

**DOI:** 10.1186/s13027-019-0224-y

**Published:** 2019-03-05

**Authors:** Asanori Kiyuna, Taro Ikegami, Takayuki Uehara, Hitoshi Hirakawa, Shinya Agena, Jin Uezato, Shunsuke Kondo, Yukashi Yamashita, Zeyi Deng, Hiroyuki Maeda, Mikio Suzuki, Akira Ganaha

**Affiliations:** 1 Department of Otorhinolaryngology, Head and Neck Surgery, Graduate School of Medicine, University of the Ryukyus, Okinawa, 903-0215 Japan; 2 Department of Otorhinolaryngology, Head and Neck Surgery, Zhujiang Hospital, Southern Medical University, Guangzhou, China; 3 Department of Otolaryngology, Faculty of Medicine, University of Miyazaki, Miyazaki, Japan

**Keywords:** Human papillomavirus, Physical status, Viral load, p16 overexpression, Laryngeal cancer

## Abstract

**Background:**

Oropharyngeal cancers associated with high-risk type human papillomavirus (HR-HPV) infection have better prognosis than virus negative cancers. Similarly, the HPV status in laryngeal cancer (LC) may be associated with better outcome.

**Methods:**

Samples from 88 patients with LC were investigated using the polymerase chain reaction (PCR) and p16 immunohistochemistry for HR-HPV analysis. The cut-off point for p16 overexpression was diffuse (≥75%) tumor expression with at least moderate (+ 2/3) staining intensity.

**Results:**

The 5-year cumulative survival (CS) rate was 80.7% in all patients with LC. According to a combination of HR-HPV DNA status and p16 overexpression, subjects with LC were divided into four groups: HR-HPV DNA-positive/p16 overexpression-positive (*n* = 5, 5.7%; CS = 100%), HR-HPV DNA-positive/p16 overexpression-negative (*n* = 11, 12.5%; CS =81.8%), HR-HPV DNA-negative/p16 overexpression-positive (*n* = 0), and HR-HPV DNA-negative/p16 overexpression-negative (*n* = 72, 81.8%; CS = 79.5%). HR-HPV DNA-positive/p16-positive cases tended to have integrated HPV infection and high viral load, compared with HR-HPV DNA-positive/p16 overexpression-negative cases.

**Conclusions:**

LC patients with HPV infection and high levels of p16 expression might have an improved survival outcome; however, it is necessary to recruit additional LC cases with HPV infection to determine the definitive characteristics of HPV-mediated LC and estimate survival outcome. These results may contribute to the development of a useful method for selecting patients with a potentially fair response to treatment and ensure laryngeal preservation.

## Background

High-risk human papillomavirus (HR-HPV) infection has become an increasingly common cause of oropharyngeal squamous cell carcinoma (OPSCC) [1, 2]. HPV-mediated OPSCC is fairly responsive to chemoradiotherapy and has a better prognosis than HPV-unrelated OPSCC [3]. A systematic review showed that HR-HPV infection was observed in several other head and neck cancers [4]. Approximately 30% of patients with laryngeal cancer (LC) are considered to harbor HR-HPV. In a recent report, the prognosis of patients with cancer involving the oral cavity, epipharynx, and larynx was not influenced by HPV infection [5]. On the contrary, another study with a large number of patients from the United States National Cancer Database clearly showed that, among patients with cancer involving the oral cavity, hypopharynx, and larynx, those with HPV infection had a significantly better prognosis [6]. Thus, there are inconsistencies regarding disease prognosis in view of HPV infection in patients with head and neck cancer, except for OPSCC.

We previously demonstrated that both HPV DNA-positive status and p16 overexpression are useful for detecting transcriptionally active HPV infection in head and neck cancer [7]. HPV infection is commonly observed in the head and neck region, but some patients with HPV infection in this region do not overexpress p16, even in OPSCC [8, 9]. Although the rates of HPV infection vary in different head and neck regions, these patients usually harbor a small viral load. The clinical practice guidelines of the American Society of Clinical Oncology recommend that pathologists should not examine HR-HPV status routinely, except for squamous cell carcinoma of the oropharynx [10]. Similarly, p16 expression varies among head and neck regions [7, 11]. Different patterns of nuclear and cytoplasmic staining of p16 protein may also become an independent indicator of survival outside of the oropharynx [12]. Previous systematic reviews of HPV infection in LC have used inconsistent methodologies to determine HPV infection [13, 14]. Further research is required to understand the prognostic consequence of p16 overexpression and HR-HPV infection outside of the oropharynx [15]. The 8th edition of the American Joint Committee on Cancer (AJCC) Staging Manual adopted p16 immunohistochemistry findings as a surrogate marker for transcriptionally active HPV infection [16, 17]. The cutoff point for p16 overexpression is diffuse (≥75%) tumor expression, with at least moderate (+ 2/3) staining intensity. However, it remains unclear whether these criteria could be adapted for other head and neck cancers. In addition, the use of fresh frozen samples is recommended for the detection of HPV mRNA compared with routine HPV DNA detection, which uses formalin-fixed, paraffin-embedded (FFPE) samples [18].

The aim of this study was to clarify the rates and clinical characteristics of LC with transcriptionally active HPV infection diagnosed by HPV-DNA testing and p16 immunohistochemistry.

## Methods

### Subjects

This study involved 88 treatment-naïve LC patients without distant metastasis. All patients were diagnosed with laryngeal squamous cell carcinoma by pathological examination of samples and were treated at the Department of Otorhinolaryngology, Head and Neck Surgery, University of the Ryukyus, between January 2008 and December 2017. The final prognosis of patients was judged in July 2018. Classification of the tumor, node, metastasis (TNM) stage was carried out according to the AJCC Staging Manual (7th edition). To determine the clinical stage and to detect concomitant multiple primary cancers, the patients underwent physical and endoscopic examinations of the upper gastrointestinal tract, ultrasonic inspection of the neck, computed tomography (CT), and ^18^F-fluorodeoxyglucose-positron emission tomography CT imaging.

This study was approved by the Institutional Review Board of the University of the Ryukyus and was carried out in accordance with the 1975 Declaration of Helsinki, as revised in 2008. Informed consent was obtained from all LC patients before enrollment.

### Treatment

The principal treatment for the primary lesion with curative intent was conventional radiotherapy (RT) alone or laser resection in T1, concurrent chemoradiotherapy (CCRT) in T2, CCRT or surgery in T3, and surgery in T4, regardless of the presence of HPV. Nodal lesions were managed with CCRT or neck dissection combined with resection of the primary lesion. All patients who received RT (total dose, 70 Gy) had CT-assisted three-dimensional radiation treatment planning in the treatment position with mask immobilization. The protocol for CCRT was as reported previously [19]. When the primary tumor in T3 failed to show a partial response regardless of the neck lymph node response at 39.6 Gy irradiation, the patients underwent curative surgery for the primary lesion combined with neck dissection.

### Detection of HPV status and p16 immunohistochemistry

All tissue samples from primary lesions without any radiation or chemotherapy were analyzed with the polymerase chain reaction (PCR) for HPV DNA detection using fresh frozen samples, which were obtained at biopsy or surgical resection, and p16 immunohistochemistry using FFPE samples. Cases with negative HPV detection results on PCR and p16 overexpression (≥75% positive cells and at least moderate staining intensity) were further evaluated for HPV status using in situ hybridization (ISH) with HPV DNA probes (DNA ISH) [16]. Quantitative real-time PCR (qPCR) for viral load and physical status, as described below, was carried out in cases harboring HPV-16 [20, 21].

### PCR for HPV DNA detection

In brief, DNA was isolated from the tumor samples using a Gentra Puregene Tissue Kit (QIAGEN, Germantown, MD). The presence and integrity of DNA were verified in all samples by PCR β-globin gene amplification using the primers PC04 and GH20 [22]. The general consensus primer sets GP5+/GP6+ and MY09/MY11 were used to analyze the presence of HPV DNA by PCR (Table 1) as described previously [8, 16]. In addition, DNA samples negative for GP5+/GP6+ or MY09/MY11 PCR were re-amplified using a nested PCR approach with the GP5+/GP6+ primer pair. PCR products of the expected size (GP5+/GP6+, 150 bp; MY09/MY11, 450 bp) were purified and sequenced directly with an ABI PRISM 3130xl Genetic Analyzer (Applied Biosystems, Foster City, CA). The sequences were aligned and compared using the BLAST program with those of known HPV types in the GenBank database.

**Table 1:** Primers used in this study

PCR primer	Sequence (5′–3′)
GP5+	TTTGTTACTGTGGTAGATACTAC
GP6+	GAAAAATAAACTGTAAATCATATTC
MY09	CGTCCMARRGGAWACTGATC
MY11	GCMCAGGGWCATAAYAATGG
PC04	CAACTTCATCCACGTTCACC
GH20	GAAGAGCCAAGGACAGGTAC
Real-time PCR primer and TaqMan probe	Sequence (5′–3′)
E2-F	AACGAAGTATCCTCTCCTGAAATTATTAG
E2-R	CCAAGGCGACGGCTTTG
E2-Probe	FAM-CACCCCGCCGCGACCCATA-TAMRA
E6-F	GAGAACTGCAATGTTTCAGGACC
E6-R	TGTATAGTTGTTTGCAGCTCTGTGC
E6-Probe	FAM-CAGGAGCGACCCAGAAAGTTACCACAGTT-TAMRA
β-globin-F	TGGGTTTCTGATAGGCACTGACT
β-globin-R	AACAGCATCAGGAGTGGACAGAT
β-globin-Probe	FAM-TCTACCCTTGGACCCAGAGGTTCTTTGAGT-TAMRA
β-actin-F	GCGAGAAGATGACCCAGATC
β-actin-R	CCAGTGGTACGGCCAGAGG
β-actin-Probe	FAM-CCAGCCATGTACGTTGCTATCCAGGC-TAMRA

*F* Forward; *R* Reverse

### p16 immunohistochemistry

Immunohistochemistry for p16 was performed using a CINTec® p16 Histology Kit (MTM Laboratories; Roche Applied Sciences, Penzberg, Germany) according to the manufacturer’s protocol [7]. Immunolabeling was visualized by incubation in 3,3′-diaminobenzidine and stained slides were counterstained with hematoxylin.

The scoring criteria for p16 immunoreactivity used in the present study were as follows: 0, no staining; 1, 1 – < 25% of the tumor cells positive for p16; 2, 25 – < 50% positive; 3, 50 – < 75% positive; 4, ≥75% positive and weak staining intensity; and 5, ≥75% positive and at least moderate staining intensity. The term “p16 overexpression” (p16-positive) was defined as a score of 5 in the present study.

### ISH with HPV DNA probes

Biotinyl tyramide-based ISH was performed using the GenPoint™ HPV biotinylated DNA probe and the GenPoint tyramide signal amplification system for biotinylated probes according to the manufacturer’s protocol (Dako; Agilent Technologies, Inc., Santa Clara, CA). The GenPoint HPV biotinylated DNA probe reacts with HPV types 16, 18, 31, 33, 35, 39, 45, 51, 52, 56, 58, 59, and 68 in 4-μm-thick sections of FFPE samples. Detection of the hybridized probe was performed using the GenPoint detection system according to the manufacturer’s protocol, with the included primary streptavidin-horseradish peroxidase (HRP), biotinyl tyramide, secondary streptavidin-HRP, and 3,3′-diaminobenzidine l (Dako; Agilent Technologies). The slides were counterstained with hematoxylin.

### Estimation of viral load and physical status of HPV-16 by qPCR

To evaluate the viral load and physical status of HPV-16, qPCR was performed as described previously [8, 20]. Briefly, primers and TaqMan probes targeting the HPV-16 *E2* and *E6* open reading frames were used (Table 1). The primers and probes recognize the *E2* hinge region, which is deleted on HPV-16 integration. Two standard curves for the *E2* and *E6* genes were created by amplification of 10-fold serial dilutions (10^1^, 10^2^, 10^3^, 10^4^, 10^5^, and 10^6^ viral copies) of the pB-actin early plasmid, which was a gift from Karl Munger, carrying the complete HPV-16 early gene region (Addgene plasmid # 13711; Addgene, Cambridge, MA). Viral DNA load was assessed by calculating *E6* copy number. An external standard curve was created using known serial dilutions (0.3, 3, 30, and 300 ng) of human genomic placental DNA (Sigma-Aldrich; Merck KGaA, Darmstadt, Germany) for cellular DNA quantification, and β-globin was amplified as described previously. The amount of DNA was calculated by plotting the Cq values against the logarithm of the standard curve. The physical status of HPV-16 was assessed based on a previously published method [8, 21]. An *E2*/*E6* ratio ≥ 1 indicates the predominance of the episomal form, whereas a ratio of *E2* copy number/total *E6* < 1 indicates the presence of both integrated and episomal forms (mixed form).

### Detection of E6 mRNA expression by qPCR and RNA ISH in HPV-16-positive patients with p16 overexpression

*E6* mRNA expression was detected in samples from patients identified as HPV-16-positive with p16 overexpression. Total RNA was extracted from frozen tissues using RNAiso Plus (Takara Bio, Kusatu, Japan) according to the manufacturer’s instructions. Total RNA (500 ng) was used to synthesize first strand cDNA using a PrimeScript™ RT Reagent Kit with gDNA Eraser (Perfect Real Time; Takara Bio) according to the manufacturer’s instructions. To measure HPV-16 *E6* mRNA expression, qPCR was performed using the CFX96 Touch™ Real-Time PCR Detection System (Bio-Rad, Hercules, CA) and TaqMan PCR Master Mix II (Roche Molecular Systems, Pleasanton, CA). Primers and TaqMan probes (Table 1) that target the HPV-16 *E6* gene and β-actin gene were used [8, 20]. The β-actin probe was labeled with FAM at the 5′-end and with TAMRA at the 3′-end (Applied Biosystems Japan, Tokyo, Japan). The amplification conditions were: 2 min at 50 °C, 10 min at 95 °C, and a 2-step cycle of 95 °C for 15 s and 60 °C for 60 s for a total of 40 cycles. Two standard curves were generated for the *E6* and β-actin genes by amplification of serial 10-fold dilutions (10^1^, 10^2^, 10^3^, 10^4^, 10^5^, and 10^6^ copies) of the pB-actin early plasmid and the pCAG-mGFP-actin plasmid, which was a gift from Ryohei Yasuda, carrying the complete coding region of β-actin (Addgene plasmid # 21948; Addgene). The expression of the *E6* gene in each sample was normalized by the amount of the internal control β-actin.

RNA ISH for HPV-16/− 18 *E6/E7* mRNA was performed using an RNAscope® 2.5 HD Reagent Kit (Advanced Cell Diagnostics, Newark, CA) according to the manufacturer’s instructions. In brief, serial 4-μm thick sections of FFPE samples were deparaffinized in xylene and rehydrated using a graded alcohol series. Endogenous peroxidase was blocked with the RNAscope® Hydrogen Peroxide Reagent at room temperature for 10 min. Target HPV RNA retrieval was performed in RNAscope® Target Retrieval Reagent at 100 °C for 15 min. The slides were digested with RNAscope® Protease Plus at 40 °C for 30 min. The HPV-16/− 18 *E6/E7* RNA probe (RNAscope® Probe-HPV16/18) was added to the sections and a coverslip was applied. The slides were transferred to a humidified chamber for hybridization at 40 °C for 2 h. Then, the coverslips were removed and the slides were washed with RNAscope® Wash Buffer Reagent at 40 °C. Signal amplification was performed according to the manufacturer’s instructions. Detection of the hybridized probe was performed using 3,3′-diaminobenzidine (RNAscope® 2.5 HD Reagent Kit). The slides were counterstained with hematoxylin. Positive control slides (HeLa cells expressing HPV-18 *E6/E7* mRNA) were included in RNA ISH. Positive staining was identified as brown punctate dots seen in the nucleus and/or cytoplasm.

### Statistical analysis

Pearson’s chi-square test was used to compare the characteristics of LC patients according to HPV DNA and p16 overexpression status. Cumulative survival (CS) was calculated using the Kaplan-Meier method and was compared between two groups using the log-rank test. All analyses were performed with SPSS Statistical Package (SPSS, Version 25.0; SPSS, IBM Corp., Armonk, NY). *P <* 0.05 was considered significant.

## Results

### Characteristics and CS of LC patients according to HPV status

**Fig. 1:**
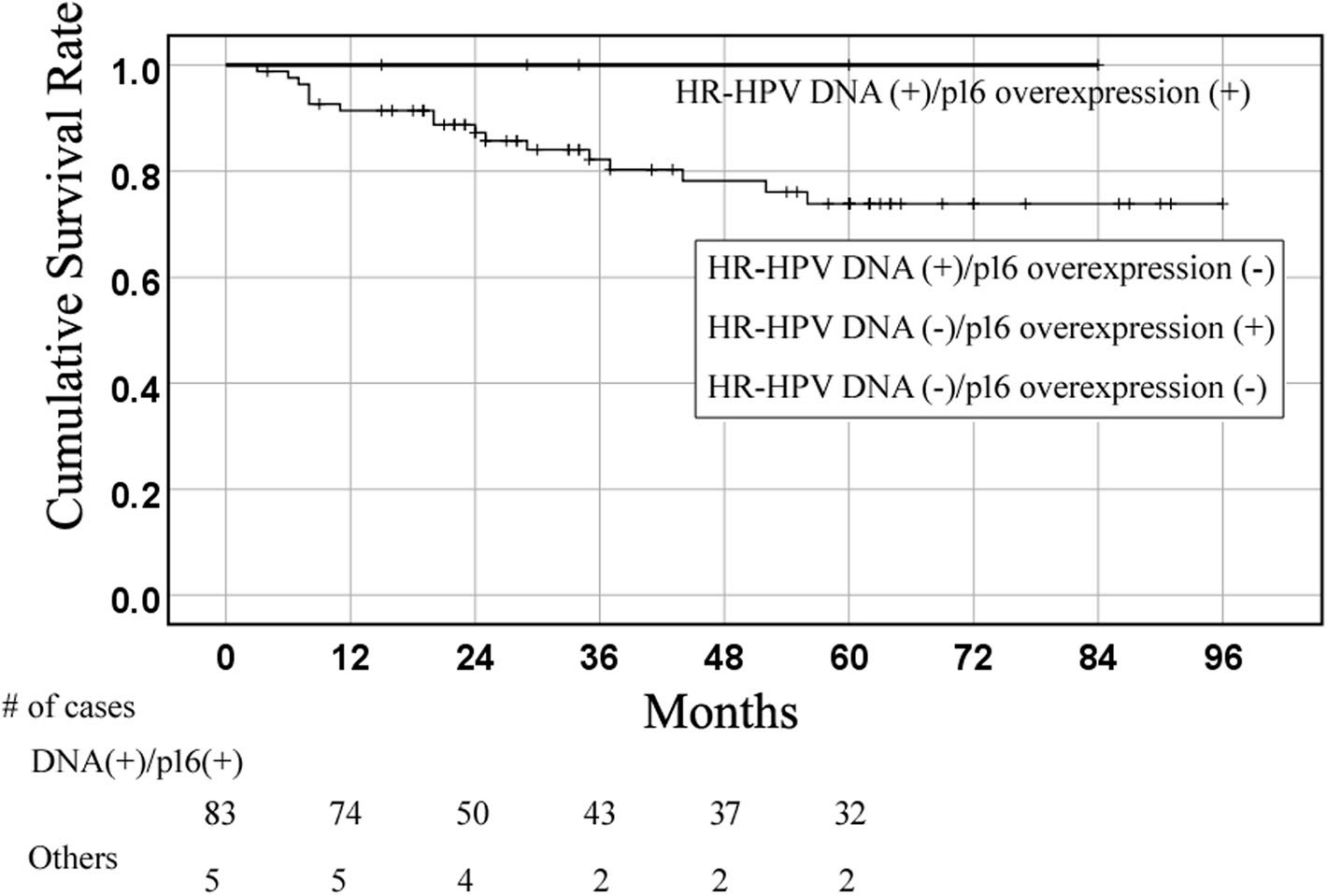
Kaplan-Meier curve of cumulative survival (CS) in laryngeal cancer (LC) Kaplan-Meier curve of CS in HPV-mediated LC (HR-HPV DNA-positive/p16 overexpression-positive) and others (HR-HPV DNA-positive/p16 overexpression-negative, HR-HPV DNA-negative/p16 overexpression-positive, and HR-HPV DNA-negative/p16 overexpression-negative).

Of the 88 patients with LC, there were 16 (18.2%) HR-HPV-positive cases, 2 (2.3%) low-risk type HPV (HPV-6)-positive cases, and 5 (5.7%) p16-positive cases. All p16-positive cases harbored HR-HPV DNA. The patients were divided into four groups according to the presence of HR-HPV DNA and p16 overexpression as follows: HR-HPV-positive/p16-positive (*n* = 5, 5.7%), HR-HPV-positive/p16-negative (*n* = 11, 12.5%), HR-HPV-negative/p16-positive (*n* = 0, 0%), and HR-HPV-negative/p16-negative (*n* = 72, 81.8%). The clinical characteristics of the patients are summarized in Table 2. The median and mean follow-up period of patients who received treatment and remained alive was 55 and 42 months, respectively. The 5-year CS rate was 80.7% in all patients with LC, 100% in HR-HPV-positive/p16-positive patients, 81.8% in HR-HPV-positive/p16-negative patients, and 79.5% in HR-HPV-negative/p16-negative patients (Fig. 1 and Table 2). There was no significant difference in CS between HPV-positive and -negative cases and between p16-positive and -negative cases. There were also no significant differences in sex, age, T category, N category, tumor subsite, clinical stage, smoking and alcohol consumption, and primary treatment among the three groups.

**Table 2:** Clinical features of LC patients by HR type HPV DNA status and p16 immunohistochemistry

	All cases (*n* = 88)	HR-HPV (+) (*n* = 16)			HR-HPV−/p16 - (*n* = 72)
			HR-HPV+/p16+ (*n* = 5)	HR-HPV+/p16- (*n* = 11)	
Sex					
Male	82	16	5	11	66
Female	6	0	0	0	6
Age (years)					
< 66	39	9	4	5	30
≥ 66	49	7	1	6	42
T					
T1, T2	63	11	3	8	52
T3, T4	25	5	2	3	20
N					
N0, N1	77	14	4	10	63
N2, N3	11	2	1	1	9
Tumor subsite					
Supraglottis	22	3	1	2	19
Glottis	59	12	4	8	47
Subglottis	7	1	0	1	6
Stage					
I, II	59	11	1	8	48
III, IV	29	5	2	3	24
Smoking habit (pack years)					
< 40	39	10	3	6	29
≥ 40	49	6	0	5	43
Alcohol consumption (g/day)					
< 40	50	9	3	5	41
≥ 40	38	7	0	6	31
Primary treatment					
Surgery±RT/CCRT CCRT to Surgery	40	6	2	4	34
RT or CCRT	48	10	1	7	38
5-year cumulative survival (%)	80.7	87.5	100	81.8	79.5

**Fig. 2:**
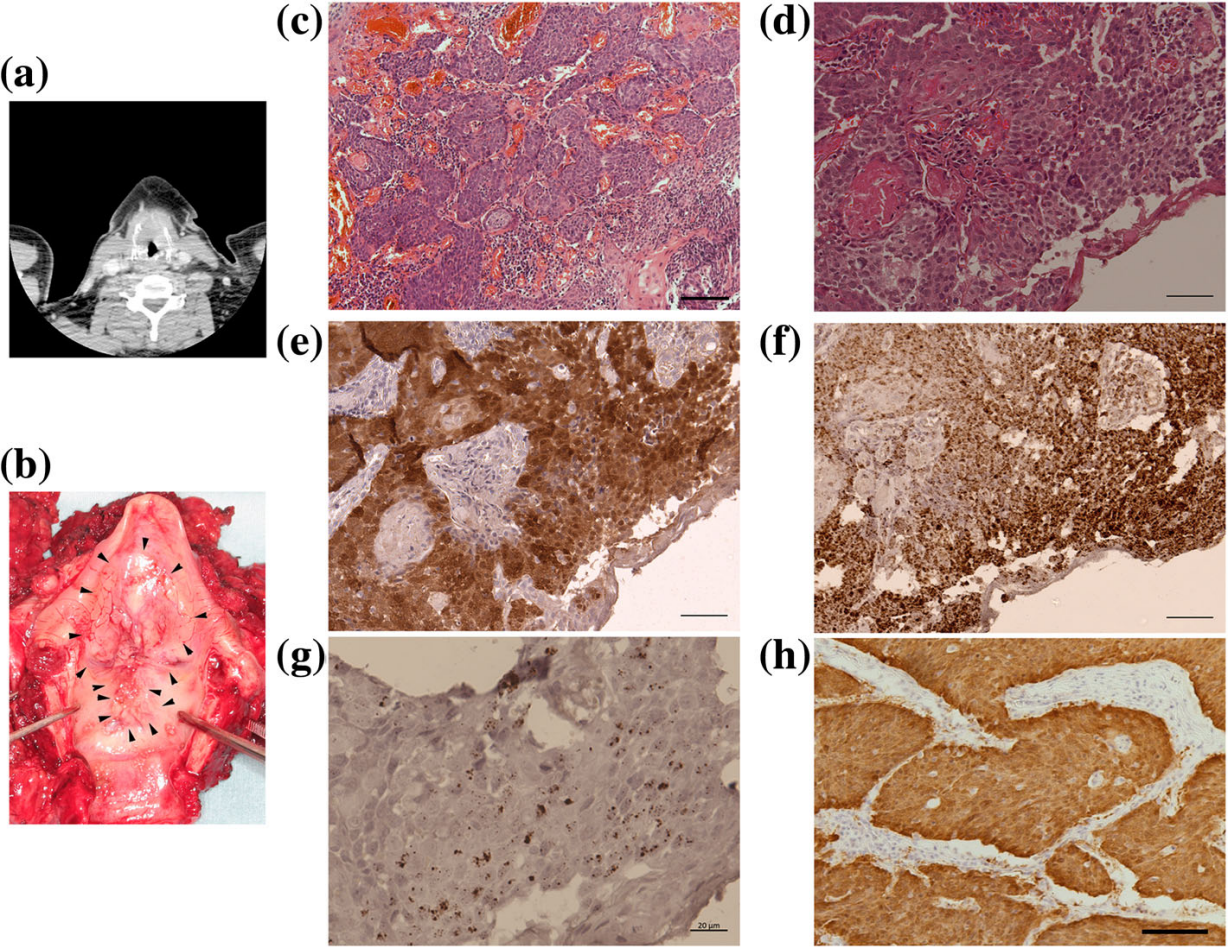
Representative case data. **a** Computed tomographic examination of the neck. The thyroid cartilage is invaded by LC. **b** Surgical specimen of the larynx. A large transglottic tumor is noted. **c**–**g** Histology results of the primary LC lesion. Hematoxylin-eosin staining at low (**c**, bar = 100 μm) and high magnification (**d**, bar = 50 μm); p16 immunohistochemical staining (**e**, bar = 50 μm); DNA in situ hybridization (ISH) (**f**, bar = 50 μm); RNA ISH (**g**, bar = 20 μm). (**h**) p16 immunohistochemical staining in a metastatic lymph node (bar = 100 μm).

Regarding the types of HR-HPV in the present study, 13 patients had HPV-16 and 1 patient had HPV-33. For the remaining 2 cases, the HPV type was indeterminate, because of HPV-positive results on DNA ISH despite negative PCR results. Only 5 of the 16 HR-HPV-positive LC cases showed p16 overexpression. The case with HPV-33 did not show p16 overexpression. Of the 13 HPV-16-positive cases, 7 were successfully analyzed to determine physical status and viral load. These cases had 0.3–7.7 × 10^4^ copies/ng genomic DNA and all samples showed complete to mixed integration. The viral load in HPV-16-positive/p16-positive cases (3 cases) was 58.4–7.7 × 10^4^ copies/ng genomic DNA, while HPV-16-positive/p16-negative cases (4 cases) had 0.3–2.1 copies/ng genomic DNA (Table 3). Although statistical analysis could not be performed because of the small number of cases, there was a large difference in viral load between the two groups. *E6* mRNA and *E6/E7* expression using qPCR and RNA ISH, respectively, were successfully investigated in 1 case in the HR-HPV-positive/p16-positive group (Fig. 2). There were insufficient samples to analyze *E6* mRNA expression in the other cases in the HR-HPV-positive/p16-positive group.

**Table 3:** Viral load and physical status in cases with HR-HPV-positive status and p16 overexpression

Case #	Age (y)	Sex	PCR	DNA ISH	Viral load (copies/ng DNA)	Integration	TN	Prognosis
1	41	M	HPV-16	+	77,735.0	Mixed	T4aN2c	Alive w/o disease,84 mo
2	41	M	HPV-16	NA	34,671.6	Mixed	T1bN0	Alive w/o disease 34 mo
3	78	M	HPV-16	NA	58.4	Mixed	T3 N1	Alive w/o disease 15 mo
4	58	M	–	+	NA	NA	T1bN0	Alive w/o disease 60 mo
5	59	M	–	+	NA	NA	T1aN0	Alive w/o disease 29 mo

#, number; y, years; NA, not available; TN, cT stage and cN stage; mo, months; w/o, without

Of the 88 cases with LC, 7 showed p16 immunoreactivity to some degree; 5 with a score of 5 (≥75% p16-positive and at least moderate staining intensity), 1 with a score of 3 (50 ≤ p16-positive < 75), and 1 with a score of 1 (1 – < 25% of the tumor cells positive for p16). Thus, all 5 patients with a score of 5 harbored HPV DNA, while 2 cases with < 75% p16 expression had no HR-HPV infection.

### Representative case of HPV-mediated LC

#### Patient data

A 41-year-old man, with a smoking history of 40 pack years and alcohol drinking of 20 g/day, presented to a referral hospital with a 1-year history of hoarseness. Physical examination revealed a transglottic tumor located mainly in the supraglottic region; CT showed a transglottic mass with invasion of the thyroid cartilage and multiple swollen lymph nodes in the neck bilaterally (Fig. 2a and b). The transglottic lesion was confirmed as moderately differentiated squamous cell carcinoma on pathologic examination (Fig. 2c and d). Thus, the diagnosis was supraglottic cancer, stage cT4N2cM0.

Total laryngectomy combined with bilateral neck dissection was performed for the LC. Pathological examination of the resected specimen revealed pT4apN2c with thyroid cartilage invasion; the surgical margins were negative. Examination of the lymph nodes revealed metastasis to 6 lymph nodes in the left side of the neck (4 at level II and 2 at level III) and 1 (level II) in the right side of the neck. CCRT after surgery was carried out with 50.4 Gy irradiation to the entire neck, and 2 cycles of nedaplatin plus 5-fluorouracil as adjuvant therapy [19]. As of 7 years since the above treatment, the patient is alive without local recurrence or distant metastasis.

#### HPV infection, viral load, and integration status

The HPV viral load and physical status of this patient are shown as Case 1 in Table 3.

PCR for HPV DNA revealed the presence of HPV-16 infection (Fig. 2f). qPCR showed that the HPV-16 viral load was 77,735 copies/ng genomic DNA and the *E2/E6* ratio was 0.11; thus, the HPV-16 integration status was judged to be mixed type. *E6* mRNA expression was also detected with RT-PCR as well as RNA ISH (Fig. 2g).

#### Immunohistochemical examination of p16 expression

Strong p16 expression was observed in metastatic lymph nodes (Fig. 2h) as well as the primary lesion (Fig. 2e).

## Discussion

There have been contradictory reports regarding the presence of HPV and p16 expression in LC [13, 14, 23]. Possible reasons for this discrepancy among studies may be because of differences in the definition of p16 expression, detection methods for HPV infection (ISH, PCR, etc.), and sample preparation methods for analysis (fresh frozen or FFPE samples) [7, 18, 23–25]. In the present study, 16 LC cases (18.2%) had HPV infection on PCR/DNA ISH analysis using fresh frozen samples. In contrast, p16 overexpression was found in only 5 of 16 patients with HPV infection. Patients with a p16 expression score of 1 or 3 did not harbor HPV DNA based on p16 immunoreactivity in the present study. On the contrary, a p16 expression score of 5 (equal to p16 overexpression) was only observed in patients with high viral load and HPV-related oncogene mRNA expression. Thus, p16 overexpression may be used as a surrogate marker in LC, as in OPSCC, although the number of cases in the present study was limited.

In this study, HPV DNA was detected only by ISH DNA, and not PCR, in 2 cases. PCR-based analysis is usually more effective for HPV detection and is cheaper than DNA ISH. There are several reasons for this outcome. One explanation is that breakpoints or deletions may have occurred in L1 at the time of HPV integration and HPV DNA only existed in the integrated form in the tumor. Another explanation is that it is difficult to obtain a sufficient amount of specimen for analysis before treatment in LC, because laryngeal cancer lesions are sometimes very small. Indeed, the clinical T stages in 2 cases were T1a and T1b, respectively (Table 3). In diagnosing HPV-mediated LC, the first step is p16 immunohistochemistry. When p16 overexpression is observed, then DNA ISH is carried out to confirm HPV infection using slides, similar to p16 immunohistochemistry. If available, PCR analysis is added to DNA ISH to determine the HR-HPV type.

A systematic review of 1668 LC patients [13] showed a prevalence of HR-HPV of 26.6%, and HPV-16 was the most frequently observed type with a prevalence 19.8%. In addition, Ndiaye et al. published a systematic review of 2739 LC cases, demonstrating that the prevalence of HR-HPV DNA was 22.1% and the prevalence of HPV-16 DNA was 13.4% [14]. The prevalence of HPV in the present study was consistent with that of these systematic reviews. However, an international cross-sectional study of 1042 LC cases using FFPE samples demonstrated that the HR-HPV positivity rate was only 5.6%, mRNA expression was 4.8%, and p16 expression (positivity in > 25% of tumor cells) was 5.6% [23]. Previous systematic reviews did not use a common definition of p16 positivity, and HPV DNA detection methods are inconsistent among studies. In the present study, we adopted an HPV analysis method previously used in OPSCC to analyze LC patients. According to the analysis of HPV DNA PCR and p16 expression in the present study, ≥75% positive cells and moderate staining intensity for p16 expression is a suitable condition for confirming HPV infection, which is similar to that of OPSCC. These cases also had high HPV-16 viral load, compared with cases with no p16 overexpression. Our results show that the prevalence of HPV-mediated LC was 5.7% in LC cases, consistent with previous studies [26, 27].

Because the rate of HPV-mediated LC is much smaller than that of OPSCC, and LC cases with T1 and T2 usually have a fair treatment response and survival outcome, analysis of HPV infection in LC may be clinically relevant in patients with advanced disease who desire laryngeal preservation. In our series, CS in HPV-mediated LC was better than in other LC cases, but there was no significant difference in CS between HPV-mediated LC and non-HPV LC, likely because the number of HPV-mediated LC cases was limited. Although several studies refute the utility of HPV testing in LC, further study is needed to clarify survival outcome with the combination of HPV DNA detection and p16 overexpression. In previous studies in OPSCC, 3–4% of cases showed p16 overexpression without HPV infection and had a worse treatment response than cases with HPV-mediated OPSCC [9, 28]. Therefore, the combination of p16 overexpression and HPV DNA analysis is reliable for the detection of HPV-mediated LC.

## Conclusions

Although the number of HPV-mediated LC cases is much smaller than that of OPSCC, several cases had carcinogenesis based on HPV infection. Survival outcome might be better for cases with HPV-mediated OPSCC; thus, the recruitment of large numbers of HPV-mediated LC cases is needed to outline definitive criteria for HPV-mediated LC. These results may contribute to developing a useful method for selecting patients with the potential for a fair response to treatment and to facilitate laryngeal preservation.

## Abbreviations

AJCC: American Joint Committee on Cancer
CCRT: concurrent chemoradiotherapy
CT: Computed tomography
LC: Laryngeal cancer
FFPE: Formalin-fixed paraffin-embedded
HPV: Human papillomavirus
HR-HPV: High-risk type HPV
ISH: In situ hybridization
OPSCC: Oropharyngeal squamous cell carcinoma.
CS: Cumulative survival
PCR: Polymerase chain reaction
qPCR: quantitative real-time PCR

## References

[1] Nasman A, Attner P, Hammarstedt L, Du J, Eriksson M, Giraud G (2009). Incidence of human papillomavirus (HPV) positive tonsillar carcinoma in Stockholm, Sweden: an epidemic of viral-induced carcinoma?. Int J Cancer.

[2] Chenevert J, Chiosea S (2012). Incidence of human papillomavirus in oropharyngeal squamous cell carcinomas: now and 50 years ago. Hum Pathol.

[3] Ang KKHJ, Wheeler R, Weber R, Rosenthal DI, Nguyen-Tan PF, Westra WJ (2010). Human papillomavirus and survival of patients with oropharyngeal carcinoma. N Engl J Med.

[4] Wagner S, Sharma SJ, Wuerdemann N, Knuth J, Reder H, Wittekindt C (2017). Human papillomavirus-related head and neck Cancer. Oncol Res Treat.

[5] Fakhry C, Westra WH, Wang SJ, van Zante A, Zhang Y, Rettig E (2017). The prognostic role of sex, race, and human papillomavirus in oropharyngeal and nonoropharyngeal head and neck squamous cell cancer. Cancer..

[6] Ko HC, Harari PM, Sacotte RM, Chen S, Wieland AM, Yu M (2017). Prognostic implications of human papillomavirus status for patients with non-oropharyngeal head and neck squamous cell carcinomas. J Cancer Res Clin Oncol.

[7] Deng Z, Hasegawa M, Aoki K, Matayoshi S, Kiyuna A, Yamashita Y (2014). A comprehensive evaluation of human papillomavirus positive status and p16INK4a overexpression as a prognostic biomarker in head and neck squamous cell carcinoma. Int J Oncol.

[8] Deng Z, Hasegawa M, Kiyuna A, Matayoshi S, Uehara T, Agena S (2013). Viral load, physical status, and E6/E7 mRNA expression of human papillomavirus in head and neck squamous cell carcinoma. Head Neck.

[9] Rietbergen MM, Brakenhoff RH, Bloemena E, Witte BI, Snijders PJ, Heideman DA (2013). Human papillomavirus detection and comorbidity: critical issues in selection of patients with oropharyngeal cancer for treatment De-escalation trials. Ann Oncol.

[10] Fakhry C, Lacchetti C, Rooper LM, Jordan RC, Rischin D, Sturgis EM (2018). Human papillomavirus testing in head and neck carcinomas: ASCO clinical practice guideline endorsement of the College of American pathologists guideline. J Clin Oncol.

[11] Yamashita Y, Hasegawa M, Deng Z, Maeda H, Kondo S, Kyuna A (2015). Human papillomavirus infection and immunohistochemical expression of cell cycle proteins pRb, p53, and p16(INK4a) in sinonasal diseases. Infect Agent Cancer.

[12] Zhao N, Ang MK, Yin XY, Patel MR, Fritchie K, Thorne L (2012). Different cellular p16^INK4a^ localization may signal differenct survival outcomes in head and neck cancer. Br J Cancer.

[13] Li X, Gao L, Li H, Gao J, Yang Y, Zhou F (2013). Human papillomavirus infection and laryngeal cancer risk: a systematic review and meta-analysis. J Infect Dis.

[14] Ndiaye C, Mena M, Alemany L, Arbyn M, Castellsagué X, Laporte L (2014). HPV DNA, E6/E7 mRNA, and p16INK4a detection in head and neck cancers: a systematic review and meta-analysis. The Lancet Oncology.

[15] Vitzthum LK, Mell LK. The role of p16 as a biomarker in nonoropharyngeal head and neck cancer. Oncotarget. 2018;(70):33247–8. 10.18632/oncotarget.26053 PMC6161792 30279955

[16] O'Sullivan B, Huang SH, Su J, Garden AS, Sturgis EM, Dahlstrom K (2016). Development and validation of a staging system for HPV-related oropharyngeal cancer by the international collaboration on oropharyngeal cancer network for staging (ICON-S): a multicentre cohort study. Lancet Oncol.

[17] Doescher J, Veit JA, Hoffmann TK (2017). The 8th edition of the AJCC Cancer staging manual : updates in otorhinolaryngology, head and neck surgery. HNO..

[18] Bussu F, Sali M, Gallus R, Vellone VG, Zannoni GF, Autorino R (2013). HPV infection in squamous cell carcinomas arising from different mucosal sites of the head and neck region. Is p16 immunohistochemistry a reliable surrogate marker?. Br J Cancer.

[19] Hasegawa M, Maeda H, Deng Z, Kiyuna A, Ganaha A, Yamashita Y (2014). Prediction of concurrent chemoradiotherapy outcome in advanced oropharyngeal cancer. Int J Oncol.

[20] Ikegami T, Uehara T, Deng Z, Kondo S, Maeda H, Kiyuna A (2018). Detection of human papillomavirus in branchial cleft cysts. Oncol Lett.

[21] Peitsaro P, Johansson B, Syrjanen S (2002). Integrated human papillomavirus type 16 is frequently found in cervical cancer precursors as demonstrated by a novel quantitative real-time PCR technique. J Clin Microbiol.

[22] Saiki RK, Scharf S, Faloona F, Mullis KB, Horn GT, Erlich HA (1982). Enzymatic amplification of beta-globin genomic sequences and restiriction site analysis for diagnosis of sicle cell anemia. Science.

[23] Castellsague X, Alemany L, Quer M, Halec G, Quiros B, Tous S (2016). HPV involvement in head and neck cancers: comprehensive assessment of biomarkers in 3680 patients. J Natl Cancer Inst.

[24] Melkane AE, Mirghani H, Auperin A, Saulnier P, Lacroix L, Vielh P (2014). HPV-related oropharyngeal squamous cell carcinomas: a comparison between three diagnostic approaches. Am J Otolaryngol.

[25] Gronhoj Larsen C, Gyldenlove M, Jensen DH, Therkildsen MH, Kiss K (2014). Correlation between human papillomavirus and p16 overexpression in oropharyngeal tumours: a systematic review. Br J Cancer.

[26] Halec G, Holzinger D, Schmitt M, Flechtenmacher C, Dyckhoff G, Lloveras B (2013). Biological evidence for a causal role of HPV16 in a small fraction of laryngeal squamous cell carcinoma. Br J Cancer.

[27] Young RJ, Urban D, Angel C, Corry J, Lyons B, Vallance N (2015). Frequency and prognostic significance of p16(INK4A) protein overexpression and transcriptionally active human papillomavirus infection in laryngeal squamous cell carcinoma. Br J Cancer.

[28] Mena M, Taberna M, Tous S, Marquez S, Clavero O, Quiros B (2018). Double positivity for HPV-DNA/p16(ink4a) is the biomarker with strongest diagnostic accuracy and prognostic value for human papillomavirus related oropharyngeal cancer patients. Oral Oncol.

